# High-Cutoff Hemodialysis Therapy for Patients with Light Chain Cast Nephropathy and AKI Requiring Dialysis: Commentary

**DOI:** 10.34067/KID.0000000000000149

**Published:** 2023-05-22

**Authors:** Laura Cosmai, Maurizio Gallieni

**Affiliations:** 1 Nephrology and Dialysis Unit, ASST Fatebenefratelli Sacco, Milan, Italy; 2 Department of Biomedical and Clinical Sciences, Università degli Studi di Milano, Milan, Italy

**Keywords:** dialysis, cast nephropathy, hemodialysis, high cutoff, light chains, membrane, membrane, treatment

AKI or chronic kidney injury occurs in approximately half of the patients with multiple myeloma. AKI represents a significant predictor of poor prognosis of kidney function recovery and survival if compared with patients with multiple myeloma without kidney damage. A considerable number of patients develop severe kidney impairment, which requires dialysis. In patients with light chain cast nephropathy, the need for dialysis increases to 50%. Independence from dialysis and better outcomes have been associated with treatments leading to a 50% decrease in free light chain (FLC) levels.^1^

Cast nephropathy is the leading cause of AKI: Overproduction of FLCs by malignant plasma cells leads to precipitation in the distal tubules; furthermore, the increased proximal tubule reabsorption of FLCs causes the activation of tubule-interstitial inflammatory and profibrotic pathways, promoting direct tubular toxicity and renal fibrosis.

Early reduction of FLC burden has become a key treatment target to improve kidney outcomes. Removing FLCs by extracorporeal techniques could be effective until antimyeloma drugs have a therapeutic effect. The evolution of multiple myeloma therapy in recent decades, specifically proteasome inhibitors, immunomodulatory imide drugs, and anti-CD38 monoclonal antibodies, led to a significantly improved hematological response rate and survival; moreover, these regimens have changed kidney outcomes, acutely reducing FLC concentrations by plasma cell depletion, which normally takes days to weeks.

There are no guidelines available to determine the approach for the early removal of FLCs. In theory, high-cutoff hemodialysis (HCO HD), using a membrane with large pores (from 0.008 to 0.01 *μ*m and a 45–60 kDa cutoff) to remove FLCs, could be combined with effective chemotherapy to avoid AKI and ESKD. However, the prognostic role of HCO HD on renal outcomes and its relationship with novel multiple myeloma therapy is still controversial. A pro–con debate in this issue of *Kidney360*^2,3^ provides an overview of the available data on whether HCO HD can provide improved clinical benefits.

The efficient role in the clearance of FLCs by a HCO dialyzer emerged from studies by Hutchison *et al.*,^4^ showing a promising *in vitro* clearance of approximately 90% of FLCs over 3 weeks, with clearance rates ranging from 9 to 30 ml/min. Over time, the interest in HCO HD was raised by subsequent case series, *in vivo* studies, and research that achieved high rates of FLC removal and hemodialysis independence. Xing *et al.*^5^ revised the issue, reporting a removal rate of FLCs of approximately 27%–75% from HCO HD sessions, with 42%–86% of patients recovering renal function and becoming hemodialysis-independent.

Thus, two randomized controlled trials, the MYRE and the EuLite studies,^6,7^ were designed to investigate HCO HD compared with conventional high-flux hemodialysis. The results from both studies were disappointing, failing to reach a statistically significant dialysis-independent rate in the first 3 months compared with the control group.

In this complex scenario, does it make sense to use HCO HD as renoprotective therapy in addition to hematological treatments until their therapeutic effect on plasma cells?

Latcha and Leung, the PRO contenders of this debate, discuss several potential limitations of the two randomized trials. Their critical analysis recognizes how several weak points could have influenced the achievement of the primary end point, suggesting a possible role of HCO HD in those with dialysis requiring AKI. Particularly, the MYRE primary end point, *i.e.*, a dialysis independence rate of 60% in the HCO HD group, was based on previous clinical studies without a comparator arm that did not include newer antimyeloma therapies. The current treatments alone, used in the MYRE and EuLite trials, can obtain a dialysis independence rate close to 50%. Thus, the MYRE and EuLite trials, which included 98 and 90 patients, respectively, may have needed to be more effectively designed and were likely underpowered to detect the additional positive effects of HCO HD properly.

The MYRE trial may also have allowed insufficient time to achieve the primary end point considering that dialysis independence was significantly increased in the HCO HD group at 6 months (56.5% versus 35.4%) and at 12 months (60.9% versus 37.5%). Other factors observed more frequently in the HCO HD arm, such as infections, hypercalcemia, hypovolemia, and nephrotoxic drugs, could have affected the kidneys at baseline presentation of myeloma. Contrarily, some of them could be associated with using HCO HD membranes, which could also cause significant albumin loss and hemodynamic instability.

For the EuLite trial, the PRO authors reveal some limitations in the study design and intervention, potentially affecting outcomes, including the increase of mortality reported at 24 months in the HCO HD arm. Reversible causes of AKI were not identified and treated before randomization, including inappropriate patients who may not have required dialysis.

On the CON side of this analysis, Karakala and Juncos debate the efficacy of extracorporeal FLC removal: They focus on the primary outcomes of failure of survival and dialysis independence at 3 months in the HCO HD group of Multiple Myeloma and Renal Failure study (MYRE) and EuLite trials, not supporting the use of HCO HD in addition to antimyeloma drugs. They also raise the safety issues of mortality, severe infectious adverse events reported in the HCO HD group, and concerns of a potentially lower response to chemotherapy agents with HCO HD FLC removal. Finally, lowering FLCs from HCO HD could make assessing hematological treatment failure and consent treatment modifications challenging.

From the PRO and CON debate, the benefits of HCO HD in AKI patients with multiple myeloma requiring dialysis remain controversial. Several limitations and concomitant factors contributed to the failure of the best available randomized controlled trials. To date, data suggest that the extracorporeal approach should only be used in clinical trials and should not delay the initiation of effective antimyeloma therapy with the crucial aim of plasma cell depletion and control of the production of FLCs. In agreement with the CON side, we conclude that HCO HD may not have a clear therapeutic or prognostic role in addition to modern hematological treatments, specifically in patients with kidney impairment not requiring dialysis. We emphasize the importance of a close collaboration between nephrologists and hematologists for the timely start of hemodialysis in conjunction with antimyeloma therapy, correctly identifying the subset of patients with AKI requiring dialysis. However, supporting the call by Latcha and Leung, we agree that further investigation of the potential role of HCO HD combined with antimyeloma therapy could be performed only through the conduction of adequately powered randomized clinical trials, including modern therapeutic regimens, with further attention to treat AKI due to reversible prerenal causes and the use of albumin supplementation and prophylactic antibiotics. The question remains open on the cost of extensive interventional studies when the underpowered MYRE and EuLite trials failed and suggested the potential for harm.

With the desirable advancements in dialysis techniques, more data can become available to re-evaluate the role of dialysis in cast nephropathy outcomes. In addition to HCO HD membranes, other tools are available: medium-cutoff (MCO) membranes with larger pores, the adsorbent polymethylmethacrylate membrane, and the hemodiafiltration with ultrafiltrate regeneration by adsorption in resin, which can improve the clearance of middle molecules like FLCs without serum albumin wasting. The REMOVAL-HD study^8^ has recently evaluated the safety and efficacy issues of MCO compared with high-flux dialysis; MCO membranes did not result in a significant decrease in serum albumin; and a reduction in the FLC level was reported after 2 weeks of treatment along the study intervention. These perceived clinical benefits of using MCO hemodialysis for AKI patients with multiple myeloma should be further assessed in randomized clinical trials, compared with HCO HD.

At the time of the MYRE and EuLite trials, we reached a similar conclusion.^9^ Contrarily, the International Kidney and Monoclonal Gammopathy Research Group,^10^ which addressed the issue in an extensive review article on AKI management in symptomatic multiple myeloma, suggests that in patients requiring dialysis, the combination of chemotherapy with FLC removal through HCO HD may increase renal response recovery rates, despite the controversial results from the MYRE and EuLite studies.

Considering the patients' burden, cost, and potential risks of hemodialysis, we have a clear indication against the use of HCO HD in patients with AKI who do not require kidney replacement therapy. In those needing dialysis, the question remains whether adding a more efficient membrane, with a limited cost, can give additional chances of dialysis independence. Because of potential side effects associated with the loss of albumin and other proteins, a control group with conventional hemodialysis should always be included in future trials.
